# Risk Factors for Postoperative Hemorrhage Following Thyroid Surgery: Results of a Case–Control Study and Development of a Stratified Risk Model Using Random Forest Analysis

**DOI:** 10.3390/jcm15145396

**Published:** 2026-07-09

**Authors:** Constantin Smaxwil, Ali Naddaf, Mirjam Busch, Joachim Wagner, Miriam Probst, Katharina Schiffer, Jasmin Al Hammoud, Ulrike Valina, Moritz Senne, Simone Harsch, Stefan Schopf, Ulrich Wirth, Amra Pepic, Antonia Zapf, Andreas Zielke

**Affiliations:** 1 Department of Endocrine Surgery, Endocrine Centre Stuttgart, Diakonie-Klinikum Stuttgart, 70176 Stuttgart, Germany; 2 Department of General, Visceral and Transplant-Surgery, University of Tübingen, 72076 Tubingen, Germany; 3 Outcomes Research Unit, Endocrine Centre Stuttgart, Diakonie-Klinikum Stuttgart, 70176 Stuttgart, Germany; 4 Department of General, Visceral, Endocrine, and Thoracic Surgery, InnKlinikum Mühldorf, 84453 Mühldorf am Inn, Germany; 5 Department of General, Visceral, and Transplantation Surgery, LMU University Hospital, LMU Munich, 81377 Munich, Germany; 6 Institute of Medical Biometry and Epidemiology, University Medical Center Hamburg-Eppendorf, 20251 Hamburg, Germany

**Keywords:** thyroid surgery, postoperative complication, postoperative hemorrhage, thyroidectomy, risk assessment, machine learning, prognostic score

## Abstract

**Background:** Postoperative haemorrhage (POH) is a rare but potentially life-threatening complication of thyroid surgery, with an incidence of 0.6–4%. Early identification of patients at increased risk is critical to guide perioperative management, especially in the context of evolving surgical practices and increasing demand for outpatient procedures. **Methods:** We conducted an explorative, retrospective, single-centre case–control study using a prospectively documented quality assurance dataset including 9158 thyroidectomies (2012–2019). POH requiring revision (*n* = 104) were compared to matched controls (*n* = 416; 1:4 ratio), matched by age, sex, type of procedure (uni- vs. bilateral), and year of surgery. Univariate analysis (Chi-square and *t*-test) was used to identify possible associations between candidate risk factors and POH. To supplement classical univariate statistics, we applied a Random Forest machine learning model to assess the relative importance of 25 potential variables derived from the clinical dataset based on previous literature. It was also planned to use the results to develop a proposal for a quantitative risk score, system, assigning weights to each factor (3, 1, or 0 points) depending on their relative importance for predicting POH. Patients were subsequently categorized into risk classes (low, intermediate, high) based on total point scores and reclassified. **Results:** High-impact risk factors confirmed in univariate analysis and Random Forest modelling included reoperative thyroidectomy, smoking, relevant comorbidities, medical treatment for hyperthyroidism and advanced age and were weighted with 3 points. Moderately associated variables such as regular alcohol consumption, Graves’ disease, hyperthyroid state at surgery, duration of the procedure and thyroid weight were weighted 1 point. Factors with negligible predictive value (e.g., BMI, gender, ASA classification) were assigned 0 points. The average score among patients without haemorrhage was 5.21, whereas the average score among patients with haemorrhage was 7.61. Within the matched study cohort, patients with POH accumulated higher risk scores than controls, suggesting potential discriminatory capacity. These findings formed the basis for the development of an exploratory three-stage ‘traffic light’ risk stratification model that requires external validation. **Conclusions:** A simple, interpretable point-based scoring system derived from a large matched case–control cohort identified key predictors of postoperative hemorrhage (POH) after thyroid surgery and enabled risk stratification within the study population. By combining conventional statistical methods with machine-learning approaches, the score may support individualized perioperative monitoring, surgical planning, and institutional resource allocation. However, because the model was developed using a 1:4 matched case–control design, it should be considered an exploratory risk stratification tool rather than a fully validated prediction model, and it does not directly estimate absolute POH risk or population incidence. External and prospective validation in large, representative multicentre cohorts (e.g., StuDoQ, HEDOS) is ongoing and will be required to establish calibration, generalizability, and clinical utility.

## 1. Introduction

Postoperative haemorrhage (POH) following thyroid surgery is an uncommon but serious complication, with an incidence reported between 0.6% and 4% in the literature [1,2,3]. While rare, it poses a potentially life-threatening situation due to the risk of acute airway obstruction caused by cervical hematoma formation. Mortality associated with POH, though low (0.03–0.06%), is often preventable and typically arises from delayed recognition or inadequate perioperative management [4]. The urgency and unpredictability of POH, combined with its potential consequences, make it one of the most feared complications in endocrine surgery.

Several national and international registries have highlighted the clinical impact of postoperative bleeding. For instance, a review of over 98,000 endocrine surgeries in Australia and New Zealand (ANZASM/CHASM, 2009–2020) revealed that 13 of 38 deaths following thyroidectomy were directly attributed to postoperative haemorrhage [5]. Similar findings were reported in long-term institutional data from Europe, where POH was associated with increased reoperation rates, need for tracheostomy, prolonged hospital stays, and in rare cases, mortality [6]. In our own patient cohort, the rate of revisions due to POH between the years 2002–2018 (21,341 patients) with endocrine cervical procedures was 1.04% (231 revisions), including 4 tracheostomies in cases of difficult airway and one death with intraoperative resuscitation. These outcomes underscore the need for early detection, rapid intervention, and ideally, prevention through risk stratification.

Despite the recognized importance of this complication, there is currently no widely accepted, validated predictive model for POH. Previous studies have inconsistently implicated factors such as age, male sex, hypertension, extent of surgery, Graves’ disease, and use of anticoagulants [7,8]. However, these findings are often derived from retrospective, single-arm analyses without proper control groups, and thus lack predictive precision. Moreover, many studies fail to consider complex interactions between patient-related, surgical, and disease-specific variables.

In Germany, the standard approach to thyroid surgery is an inpatient procedure due to the potential for POH. Yet with increasing pressure toward ambulatory or short-stay surgery, there is a growing need for objective tools to assess bleeding risk preoperatively. A structured, evidence-based risk model could enable tailored postoperative monitoring strategies, optimize resource allocation, and ultimately support the safe implementation of day-case thyroidectomy in selected patients. The intended purpose of the proposed model is not to replace clinical judgement but to support preoperative risk stratification and perioperative decision-making. Specifically, it is designed to assist in tailoring postoperative monitoring strategies, optimizing institutional resource allocation, and identifying patients who may be suitable candidates for short-stay or ambulatory thyroid surgery. Given its exploratory nature, the model should currently be regarded as a risk stratification tool rather than a validated prediction model, and its clinical applicability will require external prospective validation.

To address this unmet need, we conducted a comprehensive, retrospective case–control study using a large prospective quality assurance dataset. Beyond classical univariate analyses, we applied advanced machine learning techniques—specifically a Random Forest classifier—to assess the relative importance of more than 30 variables in predicting POH. Based on this, we developed a transparent and easy-to-use scoring system, assigning weighted points to each variable depending on its relative importance. The cumulative score was then used to classify patients into low-, intermediate-, and high-risk categories, forming the basis of a clinically applicable “traffic light” model.

This study aims not only to identify risk factors for POH, but also to develop an exploratory, structured risk stratification tool for identifying patients at increased risk of POH.

To our knowledge, no currently available risk model has been developed from a cohort of this size, incorporates machine-learning-based variable selection, and provides a clinically implementable risk stratification tool specifically for postoperative haemorrhage after thyroid surgery that can be used preoperatively to improve surgical planning, anticipate complications, and enhance overall patient safety.

## 2. Materials and Methods

### 2.1. Study Design and Population

This study was designed as an explorative, retrospective case–control analysis based on prospectively collected surgical quality assurance data. The data enrolled in this study were obtained from the centre’s pseudonymized quality assurance database (since 2017 the StuDoQ-Database for certified centres of Endocrine Surgery of the German Association of Surgeons, DGAV). The study was conducted by the Endocrine Centre’s certified Outcomes Research Unit and reviewed by the internal review board (IRB_001/2023), no additional clarification was required other than the consent obtained prior to entering data into the centre’s database.

All patients who underwent thyroid surgery at the Department of Endocrine Surgery, Diakonie-Klinikum Stuttgart, between January 2012 and December 2019 were screened. Out of 9158 recorded thyroid procedures, 104 patients were identified who experienced a revision-requiring postoperative haemorrhage (POH). These patients constituted the case group.

To minimize the influence of major confounding factors, a matched control group was assembled using a 1:4 case-to-control ratio. A total of 416 control cases were selected from the same surgical cohort who did not experience POH. Matching was performed based on the following criteria: sex, age range (in 5-year increments), type of surgery (unilateral or bilateral), and the year of operation. This yielded a total analytical cohort of 520 patients.

### 2.2. Data Acquisition and Variables

The following items, which could be associated with an increased risk of postoperative bleeding according to the literature review [9,10,11,12,13,14,15,16,17,18,19], were searched for in the individual case documents.

The following variables were considered as potential risk factors for POH:

Demographic data:
  Age, sex, body mass index (BMI)
Surgical parameters:
  Type of resection (uni- vs. bilateral), duration of surgery, total resected thyroid weight, primary vs. reoperative procedure, wound drainage, experience of surgeon/department
Medical history and comorbidities:
  Arterial hypertension, cardiopulmonary disease (e.g., COPD, asthma), previous myocardial infarction or stroke, coagulation disorders
Thyroid disease characteristics:
  Diagnosis of Graves’ disease, autoimmune thyroiditis, thyroid carcinoma, biochemical hyperthyroidism
Perioperative variables:
  Use of anticoagulant or antiplatelet medications during the perioperative period, preoperative dyspnoea, postoperative vomiting or hypertension
Lifestyle factors:
  Smoking and regular alcohol consumption, as documented in the preoperative assessment.
  Smoking and alcohol consumption were recorded as binary variables (yes/no) during the standardized preoperative assessment. Current tobacco users and patients reporting any regular or occasional alcohol consumption were classified as positive, whereas patients reporting no current consumption were classified as negative.

Clinical, surgical, and demographic data were extracted from the institutional database and operative records. Risk factors that were sporadic or registered as individual cases were not considered in statistical evaluation.

In addition to individual variables, subgroup patterns were analysed across age groups, sex, and type of procedure (unilateral vs. bilateral). A breakdown of POH events and controls across these strata is provided in Table 1, highlighting potential demographic trends and procedural clustering in specific patient groups.

### 2.3. Statistical and Machine Learning Analysis

Initial data analysis was conducted using standard descriptive statistics (R version 4.5.3; R Foundation for Statistical Computing, Vienna, Austria. The R packages used included tidyverse version 2.0.0, dplyr version 1.1.4, ggplot2 version 4.0.0, gtsummary version 2.5.0, arsenal version 3.6.3 and DescTools version 0.99.60.) Continuous variables were summarized using means, standard deviations, medians, and interquartile ranges, while categorical variables were reported as absolute and relative frequencies. To evaluate associations between variables and POH, univariate statistical tests were employed: Pearson’s Chi-squared test for categorical variables and Student’s *t*-test for continuous variables. As this was an exploratory analysis, *p*-values were interpreted in a descriptive manner, without correction for multiple comparisons and are not intended to indicate statistical significance.

To supplement the univariate analyses and explore the relative importance of multiple candidate risk factors for POH simultaneously, a Random Forest classifier was implemented using the ranger package in R (R Foundation for Statistical Computing, Vienna, Austria) [20]. The Random Forest analysis was performed on the matched study population. Matching variables (age, sex, type of surgery, and year of surgery) were retained as candidate predictors, whereas the matched pairs themselves were not explicitly incorporated into the Random Forest algorithm, as the objective was exploratory identification and ranking of relevant predictors rather than estimation of adjusted effect sizes. Only patients with complete information for all candidate predictors included in the Random Forest analysis were analysed (complete-case analysis). No imputation of missing values was performed. The Random Forest model consisted of 100,000 classification trees grown using bootstrap sampling with replacement. Variable importance was assessed using permutation importance, which quantifies the decrease in predictive performance after randomly permuting the values of a predictor. Higher permutation importance indicates a greater contribution of a variable to prediction, whereas values close to or below zero indicate little or no predictive contribution. Default tuning parameters of the *ranger* implementation, including the default *mtry* setting, were used. No class weighting or additional sampling strategy was applied. Model performance was assessed using the out-of-bag (OOB) error estimate. As the purpose of the Random Forest analysis was exploratory variable ranking rather than prediction model development, no separate training/test split, cross-validation, or bootstrap-based internal validation was performed.

Based on the combined output of univariate tests and Random Forest ranking, all candidate risk factors were categorized into three relevance levels:

(1) Highly relevant—consistently associated with POH (*p* < 0.05) and/or ranked top in permutation analysis.

(2) Moderately relevant—supported by the literature or borderline significance.

(3) Low or negligible relevance—not associated with POH in either method.

This classification formed the structure of the scoring system described below.

### 2.4. Development of a Weighted Risk Score

To translate analytical results into a clinically useful risk stratification tool, we constructed a pragmatic scoring system that assigned point values to each variable based on its relevance to POH prediction. Specifically, variables classified as highly relevant received 3 points, those deemed moderately relevant received 1 point, and low-relevance factors were assigned 0 points. This scoring system was designed to be both interpretable and practical for use in clinical workflows rather than a statistically optimized prediction model. The allocation of point values was intentionally pragmatic and based on a combination of Random Forest variable importance, univariate findings, and clinical judgement. The score was designed as an exploratory and easily interpretable risk stratification tool rather than a statistically optimized prediction model. Each patient’s individual score was calculated as the sum of the points assigned across all variables. The theoretical score range spanned from 0 (minimal risk constellation) to >12 (multiple high-risk features).

### 2.5. Score Distribution and Discriminatory Power

To assess the preliminary utility of the scoring system, we retrospectively applied it to all 520 patients in the study cohort. Each patient received a total score based on their individual risk profile. Patients were then categorized into three predefined risk groups:

Red: 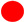 High risk

Yellow: 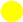 Intermediate risk

Green: 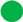 Low risk

This evaluation, tested by reclassification, served as a first test of whether the proposed model could distinguish between patients who experienced POH and those who did not. Specifically, we examined whether the distribution of total risk scores differed meaningfully between the two groups. This step was not intended as a formal validation but as a feasibility test of the model’s discriminatory capacity and potential clinical utility.

## 3. Results

The final study cohort consisted of 520 patients, including 104 individuals who experienced a postoperative haemorrhage (POH) requiring revision and 416 matched controls without any bleeding events. Matching was carried out based on sex, age group, type of surgical procedure (unilateral vs. bilateral), and year of operation. This ensured that the two groups were largely comparable in terms of their baseline characteristics.

In an additional step, we examined how age, sex, and type of procedure influenced the distribution of POH (Table 1). The majority of patients in both groups were female. It became evident that most haemorrhage events occurred in the age range between 45 and 69 years, with a slight shift toward higher age in the POH group with bilateral procedures dominating in both POH and control groups.

This demographic and procedural clustering supports the rationale for integrating both patient-related and surgical factors into a comprehensive risk model.

### 3.1. Post-Haemorrhage Risk Assessment in the Univariate Analysis

In a first step, we conducted a univariate statistical analysis to compare various clinical, surgical, and demographic parameters between the POH group and the control group. A total of 22 potential risk factors were considered. Each risk factor was assigned to one of the three groups, ‘surgery-related’, ‘patient-related’ or ‘diagnosis-related.’

Among these, several variables showed a clinically relevant association with the occurrence of postoperative haemorrhage (Table 2).

With 88.5%, the majority of POH was registered in patients with the (primary) diagnosis of “nodular goiter”; in 20.2%, a hyperthyroid metabolic state was present, and 10.6% of all cases had an autoimmune thyroid disease. POH was observed in 1% of carcinomas. Two-thirds of all POH occurred during bilateral interventions, and 8.7% during reoperation for recurrence.

In the univariate analysis, the following four risk factors were found to be relevant.

The most striking association was found for patients who underwent reoperative thyroid surgery. Within the POH group, 8.7% had undergone a reoperation, compared to only 1.9% in the control group. This difference is clinically relevant since the elevated risk is associated with repeat interventions, possibly due to altered anatomy and scar tissue formation.

A similarly robust association was observed for tobacco use. While only 17.1% of patients in the control group reported active smoking, this proportion increased to 35.6% among patients who developed postoperative bleeding (*p* < 0.001).

Furthermore, regular alcohol consumption appeared to be another relevant lifestyle-related risk factor. Patients in the POH group reported alcohol use in 41.3% of cases, compared to 25.7% in the control group (*p* < 0.002).

Another factor seems to be “relevant comorbidities” (*p* < 0.016) (summarized from cardiovascular diseases such as arterial hypertension or atrial fibrillation, renal insufficiency, metabolic diseases such as diabetes mellitus or obesity, chronic lung diseases (asthma/COPD/bronchitis), neurological diseases such as stroke, and oncological conditions). In the control group, only 43.5% had a “relevant comorbidity”, and 56.5% did not, whereas the ratio in the POH group was exactly the opposite, with 56.7% of patients having comorbidity and 43.3% without.

The use of anticoagulant medication during the perioperative period was somewhat more frequent among POH patients (10.6%) than in controls (5.8%), which is considered to be clinically relevant and consistent with findings from the previous literature. A similar result was obtained from the analysis of the main diagnosis factor (*p* = 0.082), which included nodular goiter, malignancy, and Graves’ disease as autoimmune thyroid disease.

Another factor in the group of potentially relevant risk factors was biochemical hyperthyroidism. In the POH group, 20.2% had hyperthyroidism, whereas in the control group, only 13.5% had it, resulting in a *p* = 0.084.

In contrast, several parameters showed no relevant differences between the two outcome groups.

In the surgery-related group, neither the duration of the procedure nor the resection weight showed remarkable differences between the two groups. Similarly, the primary surgical approach (unilateral vs. bilateral) was evenly distributed between groups, suggesting that extent of surgery alone may not be a decisive predictor of bleeding risk when other variables are controlled.

In the patient-related group, the individual variables of age, pulmonary disease, cerebral insult, malignant medical history, hypertension, BMI, cardiac arrhythmias, ASA, sex, coagulopathy and history of myocardial infarction showed no clinically relevant difference.

In the diagnosis-related group, medication for hyperthyroidism did not reveal any differences as a single factor.

### 3.2. Post-Haemorrhage Risk Assessment in the Random Forest Algorithm

To further explore complex variable interactions and refine risk assessment, we applied a Random Forest algorithm, which builds decision trees based on data permutations, and ranked all of the abovementioned risk variables according to their relative importance [20].

**Figure 1:**
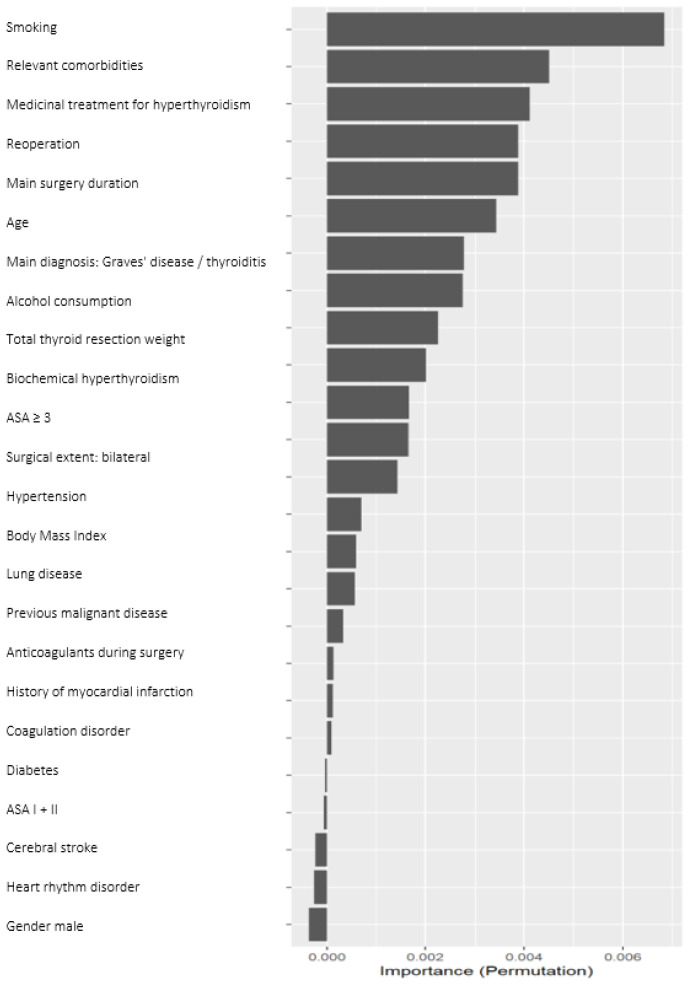
Evaluation of the random forest analysis with the indication of the respective importance (permutation) for each risk factor. The higher this value, the more important the variable. Values close to 0 indicate that the respective variable has only a random influence on the occurrence of a postoperative haemorrhage.

The Random Forest analysis (Figure 1) confirmed the previously identified high-impact factors, “reoperation” and smoking as among the top predictors. Additionally, it highlighted several other variables as highly relevant. These included “relevant comorbidities” (summarised from cardiovascular diseases such as arterial hypertension or atrial fibrillation, renal insufficiency, metabolic diseases such as diabetes mellitus or obesity, chronic lung diseases (asthma/COPD/bronchitis), neurological diseases such as stroke, and oncological conditions), medication for hyperthyroidism, duration of the procedure and age.

In contrast to the highly relevant factors, the random forest analysis identified the following as relevant factors: the presence of Graves’ disease/Thyroiditis, alcohol consumption, total thyroid resection weight and biochemical hyperthyroidism. ASA ≥ 3, bilateral surgery and hypertension were considered less relevant factors.

### 3.3. Development of a Weighted Point-Based Scoring System

Based on these findings, we developed a weighted point-based scoring system. Each variable was assigned a point value depending on its relevance: three points for highly predictive factors (e.g., reoperation, smoking), one point for moderately relevant factors (e.g., Graves’ disease, alcohol), and zero points for low- or non-predictive parameters. This scoring model was designed to be transparent, reproducible, and clinically practical.

In order to consolidate the results from both statistical and algorithmic perspectives, we compared the outputs of the univariate analysis with the feature importance rankings derived from the Random Forest classifier. The resulting risk factor assessment is illustrated in Table 3, which summarizes the degree of relevance attributed to each variable by both methods.

This dual-layered assessment combined evidence from conventional statistical analyses and Random Forest variable importance to inform the pragmatic weighting of individual risk factors within the proposed scoring system.

Although age was not statistically significant in univariate analysis, it was identified as a relevant predictor in the Random Forest analysis. For the purpose of score construction, “advanced age” was defined using the third quartile of the age distribution in the study cohort, corresponding to a cutoff of 67 years; patients aged >= 67 years were assigned 3 points.

To evaluate the discriminative potential of this scoring system, we retrospectively applied it as a reclassification to the study cohort. Each patient received a cumulative risk score based on their individual risk profile. Patients were then categorized into one of three predefined risk strata:


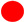
 High-risk (red category) for scores of 8 or more


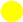
 Intermediate-risk (yellow category) for scores between 5 and 7 points


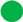
 Low-risk (green category) for scores of 4 points or below

**Figure 2:**
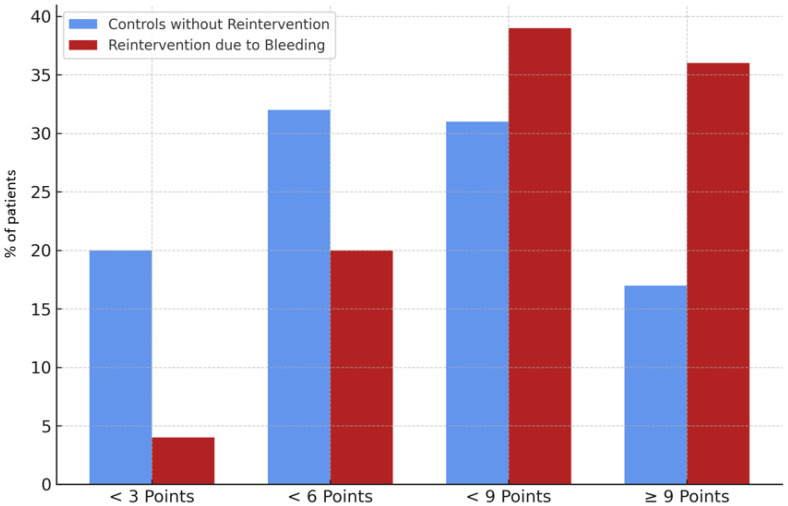
The bar chart shows in blue the patients without postoperative haemorrhage and in red the patients with postoperative haemorrhage, each assigned to the different risk groups. The strong allocation of patients with postoperative haemorrhage to the higher-risk groups is clearly recognizable. Without POH: mean 5.21 (90CI 4.9:5.5); median 5 (1Q3 3:7); with POH: mean 7.61 (90CI 7.1:8.2); median 8 (1Q3 6:12).

This internal application served as an initial test to determine whether the model was capable of distinguishing between patients with and without postoperative haemorrhage. The distribution of score points in the two groups revealed a clear trend (Figure 2), indicating that patients with POH accumulated more risk points on average than their non-bleeding counterparts. While this is not a formal validation, the observation suggests that the score may indeed possess discriminatory power and could serve as a valuable clinical decision support tool.

### 3.4. Clinical Relevance

The proposed scoring system may support preoperative risk stratification and help tailor postoperative monitoring following thyroid surgery. Patients classified as high-risk may benefit from extended observation and increased vigilance, whereas low-risk patients could be considered for short-stay or day-case surgery in appropriately selected cases. However, postoperative haemorrhage cannot be completely excluded in any patient, even in the low-risk group. Therefore, the score should be regarded as a decision-support tool that complements, rather than replaces, clinical judgement and standard postoperative surveillance.

## 4. Discussion

Postoperative haemorrhage (POH) following thyroid surgery, although relatively uncommon, remains one of the most feared complications because it can rapidly lead to potentially life-threatening airway obstruction. International registry analyses demonstrate that POH continues to contribute significantly to postoperative mortality despite modern surgical standards. The rate of postoperative bleeding is reported to be just over 1%, with approximately 70% experiencing bleeding within 6 h and only 4% after 24 h, so protocols for the early detection and management of bleeding are recommended [21]. Data from the Australian and New Zealand Audit of Surgical Mortality indicate a mortality rate between 0.03% and 0.07% following thyroid surgery [5]. In a large Spanish analysis, with a mortality rate of 0.065% after thyroid surgery, postoperative bleeding was detected as the cause of death in 30% of cases [4]. These epidemiological findings highlight the critical need for precise risk assessment to ensure early preoperative identification of patients at heightened risk.

The present study indicates that reoperations represent the strongest predictor for postoperative haemorrhage. This finding aligns closely with the established evidence. In an analysis of 30,000 operations, older age, male sex, extent of resection, and recurrence surgery were identified as risk factors for post-operative bleeding [8]. The underlying mechanisms—scar tissue formation, distortion of anatomical planes, and a more fragile vascular environment—complicate meticulous dissection and secure haemostasis. These pathophysiological considerations support the empirical observations of the present study and justify the high weighting of reoperations within the risk score. Importantly, our findings do not merely confirm reoperative surgery as a risk factor but also demonstrate its prominent contribution within a multivariable prediction framework. This observation is consistent with previous reports [8] and supports the inclusion of reoperation status as one of the highest-weighted variables in the proposed risk model.

Beyond reoperations, our analysis identifies modifiable lifestyle factors, specifically active smoking and regular alcohol consumption, as significant contributors to the risk of postoperative haemorrhage (POH). In our cohort, active smoking was more than twice as prevalent among patients who developed POH compared with controls. This is consistent with clinical evidence demonstrating that smoking is associated with an increased risk of postoperative wound complications and impaired healing, including surgical site infection and wound disruption, compared with non-smokers [22]. In a large propensity-matched cohort study encompassing over one million surgical patients, current smokers had significantly higher odds of wound disruption and surgical site complications than non-smokers, likely reflecting smoking-associated microvascular dysfunction, impaired tissue oxygenation, and disrupted inflammatory and reparative responses [23]. Regular alcohol consumption has also been linked to adverse postoperative outcomes. Systematic reviews and meta-analyses indicate that preoperative high alcohol intake is associated with an increased risk of postoperative complications, including mortality, surgical site infections, and anastomotic leakage in gastrointestinal surgery. Specifically, patients with clearly defined high alcohol consumption (>14 units/week or equivalent) had elevated odds of both infectious and surgical complications compared with non-drinkers [24]. The adverse effects of chronic alcohol use in the surgical context are biologically plausible: alcohol can impair platelet function and coagulation pathways, disrupt immune responses, and compromise vascular and endothelial integrity, thereby increasing bleeding risk and infection susceptibility. These findings underscore the need for a more comprehensive incorporation of lifestyle-related elements into preoperative risk assessment, which is often insufficiently emphasized in current clinical practice. While smoking and alcohol consumption have been associated with adverse surgical outcomes in various specialties [22,23,24], they are only inconsistently included in previously proposed thyroid surgery risk assessments. Our findings suggest that these modifiable lifestyle factors may contribute more substantially to POH risk than traditionally assumed and therefore warrant greater consideration during preoperative evaluation. Relevant comorbidities also play an important role in perioperative risk. In particular, cardiovascular and chronic pulmonary diseases have been repeatedly associated with an increased likelihood of adverse postoperative outcomes. For example, cardiovascular disease and chronic pulmonary disease were independently associated with a higher risk of short-term major postoperative complications in a cohort of patients undergoing robotic-assisted radical prostatectomy [25], where both conditions remained significant predictors after multivariable adjustment, emphasizing their relevance across different surgical settings. Furthermore, cardiorespiratory comorbidity—including both cardiac and respiratory disease—has been shown to increase the overall risk of postoperative complications following major surgery, such as esophagectomy for cancer, where preoperative cardiac comorbidity elevated the risk of pulmonary complications and adverse outcomes (e.g., higher Clavien–Dindo scores) relative to patients without such comorbidities [26]. More broadly, prospective cohort data demonstrate that the presence and number of comorbid conditions are strongly correlated with postoperative adverse events, with an increasing number of comorbidities linked to both major and minor complications [27]. In the present study, more than half of the patients who experienced POH had at least one relevant preexisting condition, underscoring systemic health status as a potent clinical risk factor. Limited cardiopulmonary reserve, increased susceptibility to perioperative blood pressure fluctuations, and underlying coagulopathies offer plausible mechanistic explanations for this observation.

It should be acknowledged that the variable “relevant comorbidities” was intentionally constructed as a composite clinical parameter comprising cardiovascular, pulmonary, renal, metabolic, neurological, and oncological conditions. The rationale for this approach was to capture the overall burden of systemic disease rather than to evaluate the individual contribution of each comorbidity subtype. Given the exploratory nature of the study and the limited number of POH events, a further subdivision into individual disease categories would have substantially reduced statistical robustness. Consequently, the observed association should be interpreted as reflecting the impact of reduced physiological reserve and increased overall morbidity rather than the effect of any specific underlying condition. Future studies with larger multicentre cohorts should investigate individual comorbidity categories separately to determine whether particular disease entities contribute disproportionately to POH risk and to further refine risk stratification models. Disease-specific factors such as hyperthyroidism or autoimmune thyroid disorders exhibited a moderate association with POH. The literature on this topic is heterogeneous; however, several large clinical studies have documented that Graves’ disease, the most common cause of hyperthyroidism, is associated with a higher risk of postoperative complications, including postoperative hematoma and bleeding-related outcomes, in patients undergoing thyroid surgery. For example, in a large NSQIP-based cohort study, patients with Graves’ disease had significantly higher odds of postoperative hematoma and related complications compared with patients undergoing thyroidectomy for other indications [28]. Similarly, a nationwide inpatient analysis demonstrated that Graves’ disease was independently associated with a higher risk of hematoma requiring reoperation and other postoperative complications following total thyroidectomy [29].

In our cohort, hyperthyroidism emerged as a relevant but not dominant contributor to bleeding risk, which is largely in agreement with previous reports demonstrating an increased but moderate risk associated with Graves’ disease and autoimmune thyroid disorders [28,29].

This aligns with the broader understanding that metabolic and autoimmune thyroid conditions may influence surgical outcomes without being primary drivers of severe complications, especially when patients are appropriately optimized preoperatively and managed in high-volume surgical centres.

It is noteworthy that variables frequently discussed in previous studies—such as BMI, sex, operative duration, and resection weight—did not demonstrate associations with POH in our analysis. These findings partially contrast earlier reports, which often attributed increased risk to male sex or large gland size. The 1:4 matching strategy applied here, controlling for age, sex, procedure type, and year of surgery, likely minimized confounding factors that may have influenced prior conclusions. This suggests that some earlier associations may have reflected surrogate markers for other unmeasured or poorly controlled risk constellations rather than causal relationships. This discrepancy highlights the importance of controlling for confounding variables when evaluating risk factors for POH and may explain some of the heterogeneity reported in the existing literature.

To our knowledge, no currently available is the complementary use of a Random Forest approach alongside conventional statistical analyses. The Random Forest was not intended to develop a final prediction model but to provide an exploratory assessment of the relative importance of candidate risk factors while accounting for potential nonlinear relationships and interactions between variables. The largely consistent identification of reoperation, smoking, relevant comorbidities, and age as important predictors across both the univariate analyses and the Random Forest approach supports the robustness of these findings.

The resulting “traffic-light” risk model provides a clear, intuitive, and clinically actionable tool for stratifying patients into low-, intermediate-, and high-risk categories. This enables tailored postoperative monitoring strategies, more efficient allocation of healthcare resources, and improved patient selection for ambulatory or short-stay thyroid surgery—an increasingly relevant consideration in modern surgical care. The distinct separation of score distributions between patients with and without POH suggests that the proposed score is able to discriminate between different risk levels within the study cohort. Nonetheless, external validation remains essential to confirm generalizability across institutions and practice settings.

Several limitations should be considered when interpreting the present findings. First, although the underlying quality assurance database was collected prospectively, the current analysis was retrospective in nature and is therefore subject to the inherent limitations of retrospective studies, including the risk of residual confounding and missing variables. Second, this was a single-centre study conducted at a high-volume endocrine surgery unit, which may limit the generalizability of the results to other institutions with different patient populations and surgical practices. Third, the proposed scoring system was internally derived and tested within the same cohort and has not yet undergone external validation. Consequently, the predictive performance and applicability of the model should be confirmed in independent multicentre cohorts before routine clinical implementation can be recommended.

Furthermore, the Random Forest analysis was intended as an exploratory method for variable prioritisation rather than formal prediction model development. Consequently, no separate training/test split, cross-validation, or bootstrap-based internal validation was performed. In addition, the weighting of the proposed traffic-light score was determined pragmatically by combining clinical judgement with the results of the univariate analyses and the Random Forest variable importance ranking, rather than being directly estimated from regression coefficients or optimisation procedures. Therefore, the proposed score should be regarded as hypothesis-generating and requires external validation before routine clinical implementation.

A further limitation should be acknowledged regarding the assessment of lifestyle-related risk factors. Smoking and alcohol consumption were recorded as binary variables based on the standardized preoperative medical history and were not quantified with respect to cumulative tobacco exposure, smoking intensity, alcohol units per week, or duration of consumption. Consequently, the present analysis cannot address potential dose–response relationships or distinguish between occasional, moderate, and heavy consumption patterns. While this simplified approach was consistent with the exploratory objective of developing an easily applicable clinical risk stratification tool, future studies should evaluate more detailed exposure measures and differentiate between current, former, and heavy users to better characterize the contribution of these lifestyle factors to POH risk.

Although several studies and meta-analyses have identified risk factors for postoperative hemorrhage after thyroidectomy, most have focused on association analyses rather than risk prediction. Large registry studies and systematic reviews consistently report factors such as reoperative surgery, male sex, older age, Graves’ disease, anticoagulant therapy, and extent of surgery as contributors to POH risk [7,8,9,10,18,19]. However, no broadly adopted clinical prediction score specifically designed for POH after thyroid surgery has emerged from these data. Therefore, the present study should not be viewed as introducing entirely novel risk factors, but rather as providing a structured and clinically implementable framework that integrates established and newly highlighted predictors into a pragmatic risk stratification model. The addition of machine-learning-based variable prioritization and the explicit incorporation of modifiable lifestyle factors represent further distinguishing features of the proposed approach.

Another limitation concerns the definition of the score thresholds used to classify patients into low-, intermediate-, and high-risk groups. These cutoffs were selected pragmatically based on the observed score distribution within the study cohort and the objective of creating a simple and clinically intuitive risk stratification framework. They were not derived from formal optimization procedures and should therefore be regarded as preliminary. As with traffic-light systems in everyday practice, the proposed categories are intended to support, rather than replace, individual clinical judgement. Future validation studies should determine whether alternative thresholds improve predictive performance and clinical utility.

Taken together, this study reaffirms key determinants of postoperative haemorrhage, identifies additional modifiable contributors, and—most notably—integrates them into a novel, data-driven, and clinically practical risk assessment tool. Against the backdrop of persistent POH-associated mortality, as documented in international datasets [4,5], such a model holds particular relevance for enhancing patient safety in endocrine surgery. The findings establish an evidence-based framework that supports standardized preoperative decision-making, clarifies appropriate postoperative observation periods, and identifies patients for whom ambulatory thyroid surgery may not be safe. With planned external validation, the model has the potential to become a central instrument in risk-adapted surgical management in the future.

## 5. Conclusions

Reoperations and certain modifiable patient factors (smoking, alcohol consumption, anticoagulant use) increase the risk of POH after thyroid surgery. The proposed “traffic light” model offers a practical risk stratification tool that may improve perioperative management and patient safety. Prospective external validation and implementation in broader datasets (e.g., StuDoQ [30], HEDOS [31]) are currently under way and integration into clinical workflows is warranted.

## Figures and Tables

**Table 1:** This table summarizes the distribution of postoperative hemorrhage (POH) events and matched controls by age group, sex, and surgical approach. It illustrates that POH was more frequent in bilateral procedures and clustered in the 45–69-year age range across both sexes.

	Female				Male			
	Bilateral		Unilateral		Bilateral		Unilateral	
Age (years)	POH	Control	POH	Control	POH	Control	POH	Control
**<=39**	5	20	2	8	1	4	-	-
**40–44**	5	20	3	12	1	4	-	-
**45–49**	13	52	4	16	1	4	2	8
**50–54**	4	16	4	16	5	20	2	8
**55–59**	4	16	4	16	5	20	2	8
**60–64**	6	24	2	8	1	4	1	4
**65–69**	7	28	1	4	7	28	2	8
**=>70**	4	16	7	28	2	8	5	20

**Table 2:** The results of univariate comparisons between patients with and without postoperative haemorrhage (POH), highlighting clinically relevant associations for reoperations, smoking, alcohol consumption, and relevant comorbidities (summarised from cardiovascular diseases such as arterial hypertension or atrial fibrillation, renal insufficiency, metabolic diseases such as diabetes mellitus or obesity, chronic lung diseases (asthma/COPD/bronchitis), neurological diseases such as stroke, and oncological conditions), while most other individual variables showed no relevant differences.

Factor	Parameter	Control Group (N = 416)	POH Cases (N = 104)	*p*-Value
**surgery-related**	Reoperation			**<0.001**
	–No	408 (98.1%)	95 (91.3%)	
	–Yes	8 (1.9%)	9 (8.7%)	
	Thyroid Weight (g)			0.328
	–Mean (SD)	61.5 (63.4)	68.3 (63.3)	
	–Median (Q1, Q3)	44.0 (23.0, 77.2)	50.0 (29.0, 83.8)	
	–Range	4.0–559.0	8.0–410.0	
	Extent of Resection			0.815
	–Bilateral procedure	281 (67.5%)	69 (66.3%)	
	–Unilateral procedure	135 (32.5%)	35 (33.7%)	
	Duration of Surgery (min)			0.904
	–Mean (SD)	90.2 (41.4)	89.6 (31.5)	
	–Median (Q1, Q3)	81.0 (61.8, 110.0)	88.5 (66.8, 107.2)	
	–Range	25.0–287.0	38.0–201.0	
**patient-related**	Smoking			**<0.001**
	–No	345 (82.9%)	67 (64.4%)	
	–Yes	71 (17.1%)	37 (35.6%)	
	Alcohol Consumption			**<0.002**
	–No	309 (74.3%)	61 (58.7%)	
	–Yes	107 (25.7%)	43 (41.3%)	
	Relevant comorbidities			**0.016**
	–No	235 (56.5%)	45 (43.3%)	
	–Yes	181 (43.5%)	59 (56.7%)	
	Anticoagulant Medication			0.080
	–No	392 (94.2%)	93 (89.4%)	
	–Yes	24 (5.8%)	11 (10.6%)	
	Age (years)			0.138
	–Mean (SD)	54.4 (13.0)	56.5 (12.9)	
	–Median (Q1, Q3)	53.8 (44.7, 64.5)	57.0 (46.8, 67.0)	
	–Range	18.5–86.3	19.0–79.0	
	Pulmonary disease			0.189
	–No	391 (94.0%)	94 (90.4%)	
	–Yes	25 (6.0%)	10 (9.6%)	
	Cerebral insult			0.243
	–No	408 (98.1%)	100 (96.2%)	
	–Yes	8 (1.9%)	4 (3.8%)	
	Malignant Medical History			0.268
	–No	389 (93.5%)	94 (90.4%)	
	–Yes	27 (6.5%)	10 (9.6%)	
	Hypertension			0.429
	–No	281 (67.5%)	66 (63.5%)	
	–Yes	135 (32.5%)	38 (36.5%)	
	BMI			0.508
	–Mean (SD)	27.2 (6.0)	27.7 (6.0)	
	–Median (Q1, Q3)	26.0 (23.0, 30.2)	27.0 (24.0, 30.2)	
	–Range	16.0–48.0	18.0–49.0	
	Cardiac arrythmias			0.595
	–No	390 (93.8%)	96 (92.3%)	
	–Yes	26 (6.2%)	8 (7.7%)	
	ASA			0.658
	I	44 (10.6%)	10 (9.6%)	
	II	311 (74.8%)	82 (78.8%)	
	III	61 (14.7%)	12 (11.5%)	
	Sex			0.848
	–Female	292 (70.2%)	72 (69.2%)	
	–Male	124 (29.8%)	32 (30.8%)	
	Coagulopathy			0.866
	–No	409 (98.3%)	102 (98.1%)	
	–Yes	7 (1.7%)	2 (1.9%)	
	History of myocardial infarction			0.879
	–No	407 (97.8%)	102 (98.1%)	
	–Yes	9 (2.2%)	2 (1.9%)	
**diagnosis-related**	Medication for hyperthyroidism			0.397
	–No	354 (85.1%)	85 (81.7%)	
	–Yes	62 (14.9%)	19 (18.3%)	
	Primary Diagnosis			0.082
	–Nodular goiter	341 (82.0%)	92 (88.5%)	
	–Malignancy	26 (6.2%)	1 (1.0%)	
	–Graves’ disease/AIT	49 (11.8%)	11 (10.6%)	
	Biochemical Hyperthyroidism			0.084
	–No	360 (86.5%)	83 (79.8%)	
	–Yes	56 (13.5%)	21 (20.2%)	

**Table 3:** The weighted risk factor classification based on combined univariate analysis and random forest evaluation. Risk factors that were both clinically relevant and algorithmically important received the highest weight, while others with either borderline evidence or minimal contribution to prediction were weighted accordingly. This structured classification became the basis for constructing the additive risk score used for patient stratification.

Risk Factor	Score
Reoperation	3
Smoking	3
Medical treatment for hyperthyroidism	3
Relevant comorbidities (e.g., cardiac)	3
Age (advanced)	3
Graves’ disease/thyroiditis	1
Regular alcohol use	1
Resection weight	1
Hyperthyroid state at surgery	1
Duration of surgery	1
ASA ≥ 3	0
Bilateral surgery	0
Hypertension	0
Other factors (BMI, sex, history of MI, stroke, cancer, etc.)	0

## Data Availability

Restrictions apply to the data presented in this study. Data were obtained from the StuDoQ Thyroid Quality Assurance Registry of the German Association of Surgeons (DGAVC) and may be available to third parties only upon request to the SAVC/DGAV and decision at the discretion of the DGAVC.
